# Outbreak of *Salmonella enterica* serotype Poona in infants linked to persistent *Salmonella* contamination in an infant formula manufacturing facility, France, August 2018 to February 2019

**DOI:** 10.2807/1560-7917.ES.2019.24.13.1900161

**Published:** 2019-03-28

**Authors:** Gabrielle Jones, Maria Pardos de la Gandara, Laura Herrera-Leon, Silvia Herrera-Leon, Carmen Varela Martinez, Roselyne Hureaux-Roy, Yasmine Abdallah, Athinna Nisavanh, Laetitia Fabre, Charlotte Renaudat, Joël Mossong, Wesley Mattheus, Cécile Huard, Caroline Le Borgne, Henriette de Valk, François-Xavier Weill, Nathalie Jourdan-Da Silva

**Affiliations:** 1 Santé publique France, Saint-Maurice, France; 2 Centre National de Référence des *Escherichia coli*, *Shigella* et *Salmonella*, Institut Pasteur, Paris, France; 3 National Centre for Microbiology, CIBER Epidemiologia y Salud Publica, Instituto de Salud Carlos III, Madrid, Spain; 4 National Centre for Epidemiology, CIBER Epidemiologia y Salud Publica, Instituto de Salud Carlos III, Madrid, Spain; 5 Directorate of the Ministry of Economy in charge of consumers’ affairs, Paris, France; 6 Department of Microbiology, Laboratoire National de Santé, Dudelange, Luxembourg; 7 National Reference Centre for *Salmonella* and *Shigella*, Sciensano, Brussels, Belgium; 8 Direction Santé et Aide aux Personnes, Commission Communautaire Commune, Brussels, Belgium; 9 French Ministry of Health, Paris, France

**Keywords:** *Salmonella*, powdered infant formula, outbreak, Poona, whole genome sequencing, France, persistent contamination

## Abstract

We describe a *Salmonella *Poona outbreak involving 31 infant cases in France. Following outbreak detection on 18 January 2019, consumption of rice-based infant formula manufactured at a facility in Spain was identified as the probable cause, leading to a recall on 24 January. Whole genome sequencing analysis linked present outbreak isolates to a 2010–11 *S.* Poona outbreak in Spain associated with formula manufactured in the same facility, indicating a persistent source of contamination.

## Outbreak alert

On 18 January 2019, a cluster of four isolates of *Salmonella enterica* serotype Poona (*S*. Poona) was identified in infants under 1 year of age. The isolates were received from 20–24 December 2018 at the French National Reference Centre for *Escherichia coli, Shigella* and *Salmonella* (NRC-ESS, Institut Pasteur). Initial investigations identified consumption of the same brand of rice-based powdered infant formula (Brand A formula). 

**Figure 1:**
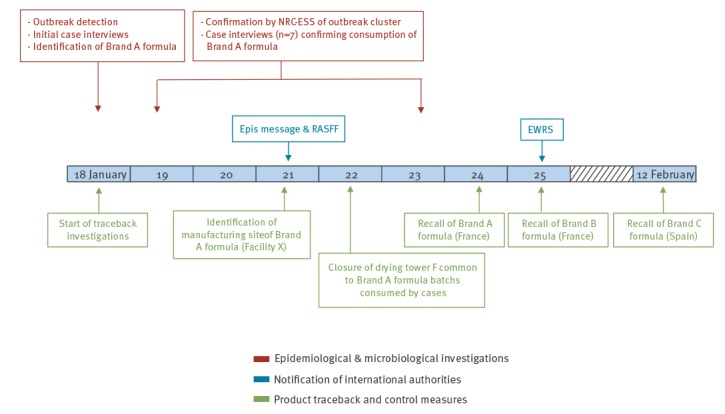
Timeline of outbreak alert and initial investigations of *Salmonella enterica* serotype Poona infections associated with consumption of infant formula, France, 18 January–12 February 2019

EPIS: Epidemic Intelligence Information System; EWRS: Early Warning and Response System: NRC-ESS: National Reference Centre-*Escherichia coli*, *Shigella* and *Salmonella*; RASFF: Rapid Alert System for Food and Feed.

On 21 January 2019, the Directorate of the Ministry of Economy in charge of consumers’ affairs (DGCCRF) identified that the Brand A formula had been manufactured at a single production facility in Spain, Facility X. After additional isolates of *S*. Poona in infants consuming Brand A formula were identified, the French distributer initiated a recall of all Brand A formula manufactured at Facility X on 24 January (Figure 1) [1]. 

Case interviews were conducted with the parents/guardians on behalf of the infant cases.

## Epidemiological investigations

For the investigation, cases were defined by Santé Publique France (SpFrance) according to the case definition in the box.

**Case definition for *Salmonella enterica* serotype Poona outbreak, France 2018–2019**

• **Confirmed case:** A laboratory-confirmed *S. *Poona infection in a child younger than 36 months, with date of symptom onset (or date of *Salmonella* spp. isolation) after 1 January 2018 and belonging to the outbreak cluster defined by the French National Reference Centre for *Escherichia coli*, *Shigella* and *Salmonella* using WGS^a^.

• **Possible case:** A laboratory confirmed *S*. Poona infection in a child younger than 36 months, with date of symptom onset (or date of *Salmonella* spp. isolation) after 1 January 2018 and undergoing WGS.

• Infants were excluded if they had a *S*. Poona infection confirmed by WGS as not belonging to the outbreak cluster.

WGS: whole genome sequencing.
^a ^See section on microbiological investigations for further details.

On 18 January 2019, after the initial alert, the families of three infants were interviewed by telephone by SpFrance. A standardised *Salmonella* questionnaire was used, which included questions relating to the consumption of foods and drinks e.g. infant formula, as well as methods of bottle preparation and exposure to reptiles, a known risk factor for *S*. Poona [2]. Two of the three infants had consumed different products of Brand A formula. Between 18 and 23 January, the NRC-ESS identified a total of nine *S*. Poona isolates in infants: four belonging to the same genomic cluster by single nucleotide polymorphism (SNP) analysis and core genome MLST (cgMLST) analysis and five infants with *S*. Poona isolates undergoing cluster analyses. Interviews of parents/guardians for seven of these infants (two families were unreachable) confirmed consumption of Brand A formula.

As at 28 March 2019, 30 confirmed cases and one possible case have been identified across 11 regions in France. *S*. Poona infection from suspected person-to-person transmission was identified in an older sibling (> 36 months) of a confirmed case but the child was excluded from the analysis as they did not fulfil the case definition. The median age of the 31 cases was 10 months (range: 2–28 months) including 13 girls and 18 boys. Week of *Salmonella* isolation ranged from week 34 2018 to week 8 2019. From 18 January to 13 March, the parents/guardians of 29 cases were interviewed.

**Figure 2:**
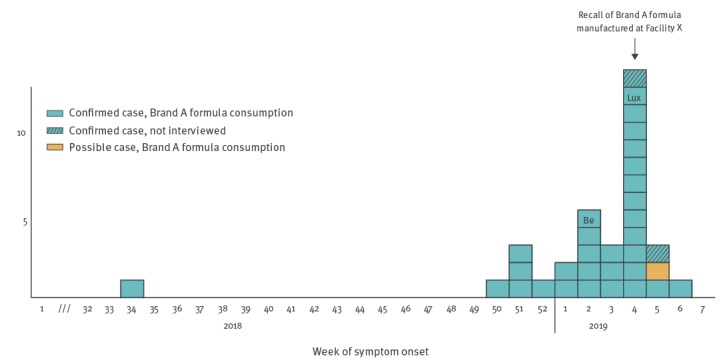
Epidemic curve of *Salmonella* Poona cases by week of symptom onset^a^, France, Belgium and Luxembourg, 2018–2019 (n = 33)

Symptom onset ranged from week 34 2018 to week 6 2019 (one case consumed formula after the recall as caretakers did not immediately receive the information) (Figure 2). No cases reported symptom onset after 8 February 2019 and the outbreak is considered over. All cases had diarrhoea, with 13 of 29 cases reporting bloody diarrhoea; 27 cases had fever (> 38°C). Thirteen cases were hospitalised, with two cases having underlying medical conditions. All infants were recovering or had recovered at the time of interviews.

Be: Belgium; Lux: Luxembourg.

^a^ Cases not interviewed are represented by week of *Salmonella* isolation.

Cases from Luxembourg and Belgium were identified following an urgent enquiry in the European Centre for Disease Prevention and Control (ECDC) Epidemic Intelligence Information System for Food- and Waterborne Diseases and Zoonoses (EPIS-FWD) posted on 21 January.

Parents/guardians all reported using Brand A formula during interviews. Rice based formula is usually recommended for lactose intolerant infants. Brand and batch numbers for formula consumed or purchased in the week before symptom onset were collected during interviews for trace back and microbiological investigations. Three different Brand A formula products were identified: first age formula for infants aged 0–6 months (two cases), second age formula for infants aged 6–12 months (standard format; 16 cases) and 6–36 months (anti-regurgitation format; 11 cases). No other common food items, including bottled water, were identified. Practices for bottle preparation were according to recommendations and did not favour bacterial proliferation.

Sixteen countries responded to an urgent enquiry in the European Centre for Disease Prevention and Control (ECDC) Epidemic Intelligence Information System for Food- and Waterborne Diseases and Zoonoses (EPIS-FWD) posted on 21 January and two additional confirmed cases were identified, one in Belgium and one in Luxembourg (ages 9 and 27 months, respectively). Both cases had consumed Brand A formula, the former purchased at a pharmacy in France and the latter on the Internet.

## Control measures and product investigations

Brand A formula was marketed in France by distributer A, who initiated a recall on 24 January of all products manufactured at Facility X. On 25 January, another French distributer of a different brand of rice-based infant formula (Brand B) recalled its products manufactured at Facility X as a precautionary measure. To date, no human cases have been linked to Brand B.

International public health authorities were informed by France of the outbreak and the distribution of Brand A formula through multiple platforms including the EPIS-FWD urgent inquiry and a Rapid Alert System for Food and Feed (RASFF: notification 2019.0224) posted on 21 January. Following the recall, an Early Warning and Response System (EWRS) message was published on 25 January.

Investigations identified seven batches of Brand A formula consumed or purchased before symptom onset for 18 cases with information available. The products were only sold in pharmacies in France by distributer A. However, distributer A also sent these products to Libya, Syria, Tunisia and Vietnam [3]. Brand A products were also distributed to European Union and European Economic Area (EU/EEA) and Morocco from online retailers or wholesaler distribution via an e-commerce site [3]. Countries with distribution or sale of the recalled Brand A formula were informed through the RASFF. The e-commerce site informed customers via email. No additional cases were reported by other countries.

Trace back investigations identified a common drying tower used by Facility X to produce the Formula A batches consumed by the cases; the tower was closed on 22 January as a precautionary measure. On 12 February, Spanish authorities recalled of a different brand (Brand C) of rice-based infant formula that had been dried in the same tower at Facility X and distributed in Spain. No *S.* Poona infections were identified in Spanish infants consuming Brand C formula.

As at 28 March, all samples from Brand A formula consumed by cases, from additional batches of Brand A formula manufactured at Facility X and from environmental samples in Facility X were negative for *Salmonella.*

## Microbiological investigations

Genomic surveillance of *Salmonella* infections has been in place at the NRC-ESS since April 2017. WGS is carried out at the Plateforme de microbiologie mutualisée (P2M) from the Pasteur International Bioresources network (PIBnet, Institut Pasteur, Paris, France) [4]. The MagNAPure 96 system (Roche Diagnostics, Indianapolis, Indiana, United States (US)) is used for DNA extraction, libraries are prepared using the Nextera XT kit (Illumina, San Diego, California, US) and sequencing is done with the NextSeq 500 system (Illumina). Serotype prediction is done by in-house scripts based on MLST [5], *fliC*, and *fljB* gene databases. Phylogenetic analysis is performed by two different approaches integrated into EnteroBase (https://enterobase.warwick.ac.uk) [6]: SNP and cgMLST analyses.

To enable rapid identification of *S*. Poona in infants and conduct timely interviews, all isolates from children under 3 years of age underwent serotyping by agglutination [7]; results were available within ca 48 hours. Following this, all identified *S*. Poona isolates underwent whole genome sequencing (WGS) in order to determine cluster affiliation.

Eighty-four *S.* Poona isolates were received at the NRC-ESS between 1 January 2018 and 15 February 2019; 69 (73.4%) of these isolates were sequenced (including all 57 *S.* Poona received since July 2018).

**Figure 3:**
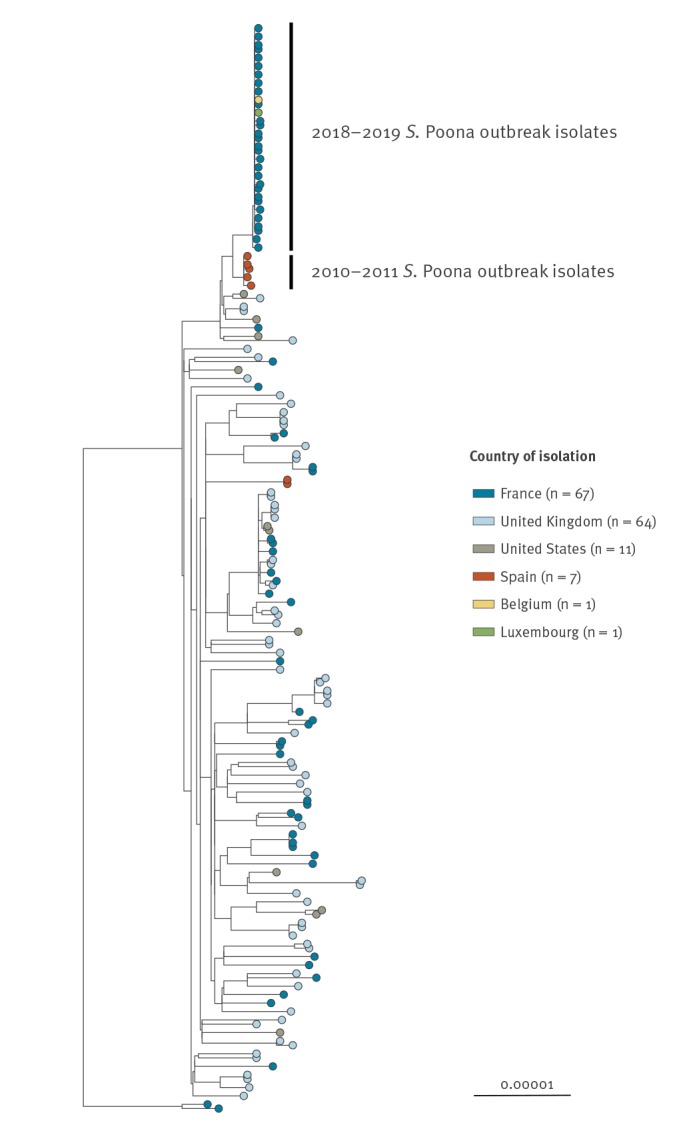
SNP-based phylogenetic tree of *Salmonella* Poona ST308 isolates performed by the NRC-ESS, France, 2019

NRC-ESS: French National Reference Centre for *Escherichia coli*, *Shigella* and *Salmonella*; S: *Salmonella*; SNP: single nt polymorphism.

The SNP analysis revealed that 31 French *S.* Poona human isolates (31/69, 45%) clustered tightly together (30 confirmed cases, one secondary transmission) (Figure 3). These clustered isolates displayed MLST type ST308 and an identical type ‘164707’ by hierarchical clustering of cgMLST data differing by five or less alleles (HC5). Two other human isolates submitted by the NRC in Belgium and the National Health Laboratory in Luxemburg also belonged to the same SNP and cgMLST HC5–164707 cluster as the French outbreak isolates (Figure 3). All studied genomes were deposited into EnteroBase and raw reads of two representative outbreak isolates (201811387 and 201900647) were also deposited to the European Nt Archive, under study accession number PRJEB31267.

The phylogenetic analysis was carried out by using the ‘create SNP project’ tool in EnteroBase on 151 genomes belonging to cgMLST type HC50–1288 with epidemic *S*. Poona isolate 201900055 used as the reference genome. The tree was based on 2,597 SNPs found in non-repetitive regions that are present in 95% or more of all the queried genomes. The tips are coloured according to the country of isolation.

Isolates from five infant and infant formula samples collected during the 2010–11 *S*. Poona outbreak in Spain were sequenced by the Spanish National Reference Laboratory (Instituto de Salud Carlos III, Madrid) and submitted to Enterobase for comparison with the French outbreak isolates. Two *S*. Poona isolates from infants in Spain collected in 2018 were also available for comparison. The cgMLST (HC20–44730) and SNP (Figure 3) analyses confirmed that the 2010–11 Spanish isolates were related to the French outbreak isolates, while the 2018 isolates were unrelated.

## Discussion

A total of 33 *S.* Poona cases were identified in this outbreak. While half of the cases were hospitalised for their salmonellosis, fortunately no serious illnesses were reported. Early detection of *S.* Poona isolates alerted SpFrance to a potential outbreak, which resulted in an immediate investigation of subsequent cases and rapid implementation of control measures, with a recall of the suspected product 6 days after outbreak detection. This outbreak illustrates the sensitivity of the French *Salmonella* surveillance system, which is based on routine WGS of human isolates received at the NRC-ESS.

In 2008, an outbreak of *S*. Give in infants in France was also linked to infant formula manufactured at Facility X [8,9]. More recently, in 2010–11, an outbreak of *S*. Poona in Spain, affecting more than 280 infants, was linked to infant formula manufactured at the same facility [10]. The brands of infant formula identified in these two outbreaks were different from Brand A. While no *Salmonella* contamination has been confirmed in Brand A formula or in environmental samples from Facility X in 2019, the results of the epidemiological investigations support the hypothesis that Brand A formula is the origin of the outbreak. Furthermore, genomic analyses confirm that the current outbreak and the 2010–11 *S*. Poona outbreak isolates are related. Investigations are ongoing at Facility X in an effort to identify the source of contamination.

Similar outbreaks linked to contamination of powdered infant formula (PIF) by a variety of *Salmonella* serotypes have been reported in several countries [10-15]. This is the fourth outbreak of *Salmonella* linked to PIF in France and the second in less than a year for which investigations strongly suggest a persistent source of *Salmonella* contamination inside a manufacturing facility [8,16,17].

The current outbreak and the *S*. Agona outbreak in 2017 in France highlight the risk of persistent *Salmonella* contamination in PIF manufacturing facilities and demonstrate the importance of WGS in identifying recurrent outbreaks. Persistent contamination in a production facility has also been reported in an outbreak linked to dry cereal [18]. PIF is not a sterile product and *Salmonella* contamination, in particular at low levels, can occur [19]. A 2008 report by the French Agency for Food, Environmental and Occupational Health and Safety (ANSES) evaluated the contamination risk for PIF and the difficulties associated with detection [20]. Low level or point source contamination, uneven distribution of *Salmonella* contamination in PIF and the efficacy of sampling plans can all impact detection [19,20]. Additionally, factors related to environmental persistence are still not fully understood. ANSES recommended that product sampling be coupled with environmental sampling as testing the product alone is not sufficient to detect potential contamination.

While in previous outbreaks in France, *S*. Agona in 2005 [17] and *S*. Give in 2008 [8,9], *Salmonella* was detected in PIF, this most recent outbreak illustrates the continuing challenge of detecting low level *Salmonella* contamination. Despite testing PIF consumed by cases as well as environmental and additional PIF samples, no *Salmonella* contamination has been detected. Understanding the limits of microbiological testing for the detection of product and environmental *Salmonella* contamination is crucial for describing the risk of persistent *Salmonella* contamination in dry food production facilities and improving detection and prevention methods.
