# Correction for Euro Surveill. 2020;24(13)

**DOI:** 10.2807/1560-7917.ES.2021.26.10.210311c2

**Published:** 2021-03-11

**Authors:** Eurosurveillance editorial team

**Affiliations:** 1 European Centre for Disease Prevention and Control (ECDC), Stockholm, Sweden

In the article ‘Outbreak of Salmonella enterica serotype Poona in infants linked to persistent Salmonella contamination in an infant formula manufacturing facility, France, August 2018 to February 2019’ by Jones et al., published on 28 March 2019, the sentence ”On 25 January, another French distributer of a different brand of rice-based infant formula (Brand B) recalled its products manufactured at Facility X as a precautionary measure” was replaced with “On 25 January, another French distributor of a different brand of dairy-based infant formula (Brand B) manufactured at Facility X in the same drying tower F recalled the product as a precautionary measure”. This change was made on 8 March 2021 on request of the authors.

